# Abdominal aortic calcification is not superior over other vascular calcification in predicting mortality in hemodialysis patients: a retrospective observational study

**DOI:** 10.1186/1471-2369-14-120

**Published:** 2013-06-05

**Authors:** Daqing Hong, Shukun Wu, Lei Pu, Fang Wang, Junru Wang, Zhengtong Wang, Hui Gao, Yue Zhang, Fei Deng, Guisen Li, Qiang He, Li Wang

**Affiliations:** 1 Division of Nephrology, Sichuan Academy of Medical Sciences and Sichuan Provincial People’s Hospital, Chengdu 610072, China; 2 Division of Nephrology, Jinhua Municipal Center Hospital, Jinhua 321000, China

**Keywords:** Vascular calcification, Mortality, Hemodialysis, Abdominal aortic calcification

## Abstract

**Background:**

KDIGO (Kidney Disease: Improving Global Outcomes) guidelines recommend that a lateral abdominal radiograph should be performed to assess vascular calcification (VC) in dialysis patients. However, abdominal aortic calcification is a prevalent finding, and it remains unclear whether other anatomical areas of VC can predict mortality more accurately.

**Methods:**

A total of 217 maintenance hemodialysis patients were enrolled at the Sichuan Provincial People’s Hospital between July 2010 and March 2011. Radiographs of the abdomen, pelvis and hands were evaluated by a radiologist to evaluate the presence of VC. The correlation between different areas of VC and all-cause or cardiovascular mortality was analyzed using univariate and multivariate models.

**Results:**

The prevalence of VC was 70.0% (152 patients), and most had abdominal aortic calcification (90.1%). During 26 ± 7 months of follow-up, 37 patients died. The VC score was independently associated with patient mortality. VC observed on abdominal radiographs (abdominal aortic calcification) was associated with all-cause mortality in models adjusted for cardiovascular risk factors (HR, 4.69; 95%CI, 1.60-13.69) and dialysis factors (HR, 3.38; 95%CI, 1.18-9.69). VC in the pelvis or hands was associated with all-cause mortality in the model adjusted for dialysis factors. When three combinations of VC in different radiographs were included in models, the presence of abdominal VC was only significantly associated with all-cause mortality in the integrated model. VC in the abdomen and pelvis was associated with all-cause mortality in the model adjusted for cardiovascular factors and the integrated model, but neither was significantly associated with cardiovascular mortality. VC in all radiographs was significantly associated with a more than 6-fold risk of all-cause mortality and a more than 5-fold risk of cardiovascular mortality compared to patients without VC.

**Conclusions:**

VC in different arteries as shown on radiographs is associated with different levels of risk for mortality. The lateral abdominal radiograph may not be superior to other radiographs for predicting patient outcomes. Further research is needed to elucidate the effects of difference burdens of VC on patient outcomes.

## Background

Cardiovascular mortality is reported to be the leading cause of mortality among patients undergoing hemodialysis [1,2]. Besides traditional risk factors for cardiovascular disease, mineral bone disorders associated with chronic renal disease, and vascular calcifications (VC) in particular, have been identified as disease-specific risk factors for cardiovascular disease (CVD) in hemodialysis patients [3-5]. In clinical observations, a strong relationship between coronary artery calcification and cardiovascular mortality has been reported in hemodialysis patients [6-9]. However, due to its high cost and radiation exposure, computed tomography (CT) is not recommended as a routine or screening technique for VC. Kidney Disease: Improving Global Outcomes (KDIGO) has recommended that a lateral abdominal radiograph and echocardiography should be used as appropriate alternatives to cardiac CT to detect VC [10]. However, as a result of the high prevalence of abdominal VC in both hemodialysis patients and the elderly population in general [11-14], its clinical value may be limited. Other vascular calcifications by radiographs have been reported, but except for coronary artery calcification,whether a more specific VC exists to predict patient outcomes than abdominal aortic calcification remains unclear.

Although a significant relationship between abdominal aortic calcification and patient mortality has been reported, most studies have described the presence of VC only in a specific artery. The mechanisms and speed of VC development, its pathophysiology, accompanying risk factors and impact on patient outcomes may differ to some degree between arteries. Few studies have analyzed the confounding effects of different regions of VC on the prognosis of hemodialysis patients. One study showed that both coronary artery calcification (HR, 3.40; 95%CI, 1.24-9.36) and aortic arch calcification (HR, 6.23; 95%CI, 1.64-23.66) remained significantly associated with mortality after they were adjusted for each other [6]. A simple scoring system to evaluate VC in hemodialysis patients has identified a positive correlation between outcomes and this VC score [15]. However, if VC in different arteries contributes differently to patient outcomes, this scoring system will need appropriate adjustment.

We hypothesized that different regions of VC have different levels of impact upon patient mortality, and that abdominal radiographs may not be the best choice for this evaluation. We analyzed lateral abdominal, frontal pelvic and bilateral hand radiographs to ascertain the degree of predictive value for patient outcomes, with particular emphasis on cardiovascular mortality.

## Methods

### Study design

This was a retrospective, single-center study of patients undergoing hemodialysis.

### Participants

We enrolled all hemodialysis patients who had received dialysis 3 times/week for >3 months. Treatment of patients with chronic kidney disease mineral bone disorders (including secondary hyperparathyroidism) included management of hyperphosphatemia by restriction of diet phosphorus intake (as recommended by a dietitian), calcium-based phosphorus binders (calcium carbonate or calcium acetate), and non-calcium phosphorus binders (sevelamer, lanthanum carbonate or temporary use of sucralfate); management of serum calcium by calcium carbonate with/without alfacalcidol/calcitriol); and management of iPTH by alfacalcidol or calcitriol. Patients with hypertension were treated with single drugs or different combinations of calcium channel blockers, beta- adrenoreceptor blockers, angiotensin converting enzyme inhibitors, and angiotensin receptor blockers. Radiographs (lateral abdominal radiograph, frontal pelvic radiograph and both hands) were taken between July 2010 and March 2011 at the Blood Purification Center in Sichuan Provincial People’s Hospital. Patients without records of laboratory investigations during this period were excluded. This study was approved by the Sichuan Academy of Medical Sciences and the Sichuan Provincial People’s Hospital Medical Ethics Committee (reference number: 2011KY-SN01). In total, 217 patients were enrolled and each signed an informed consent prior to the study.

### Measurements

**Figure 1:**
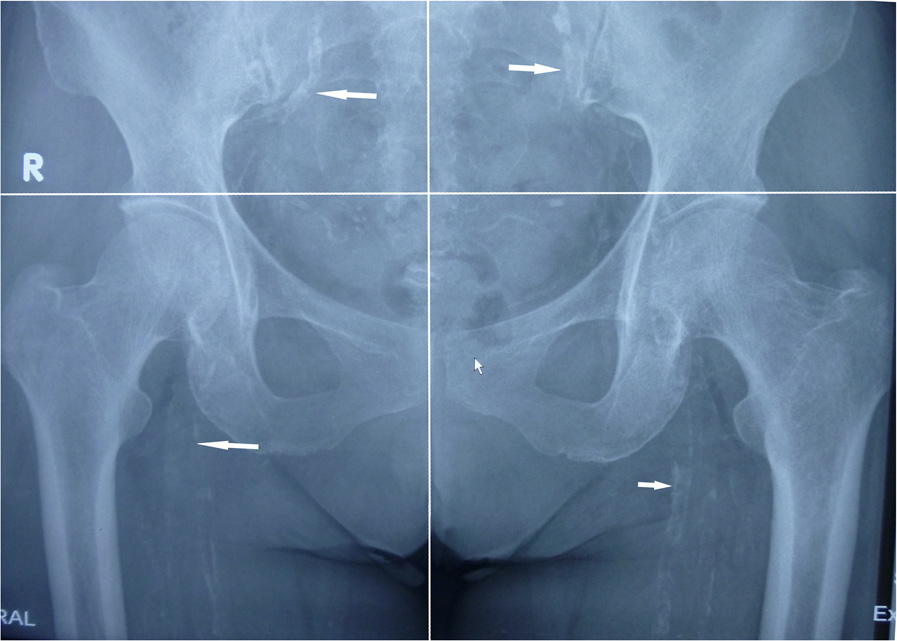
Vascular calcification evident on pelvic radiograph.

**Figure 2:**
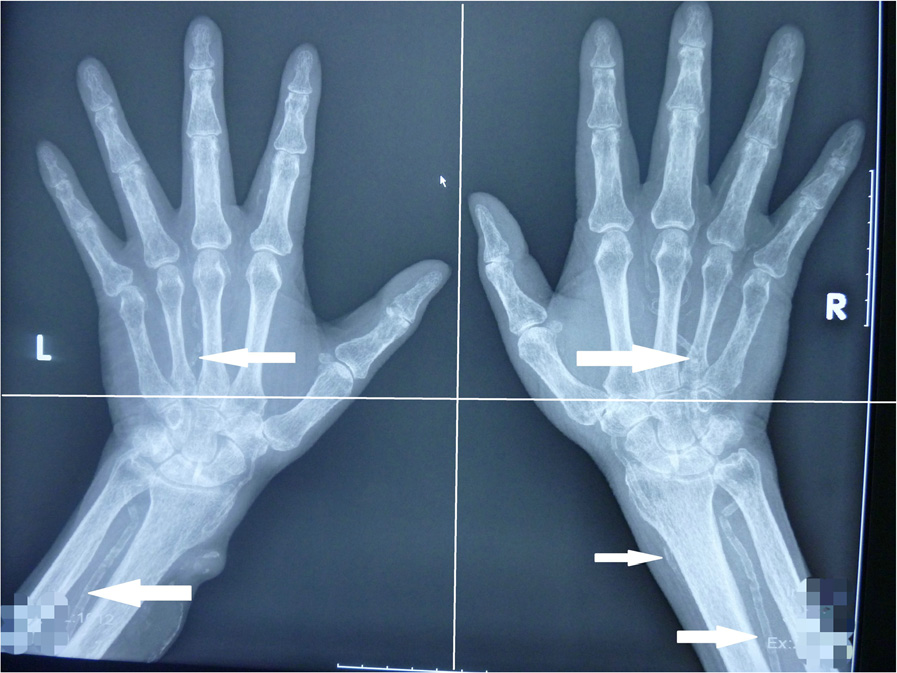
Vascular calcification in radiographs of the hand.

**Figure 3:**
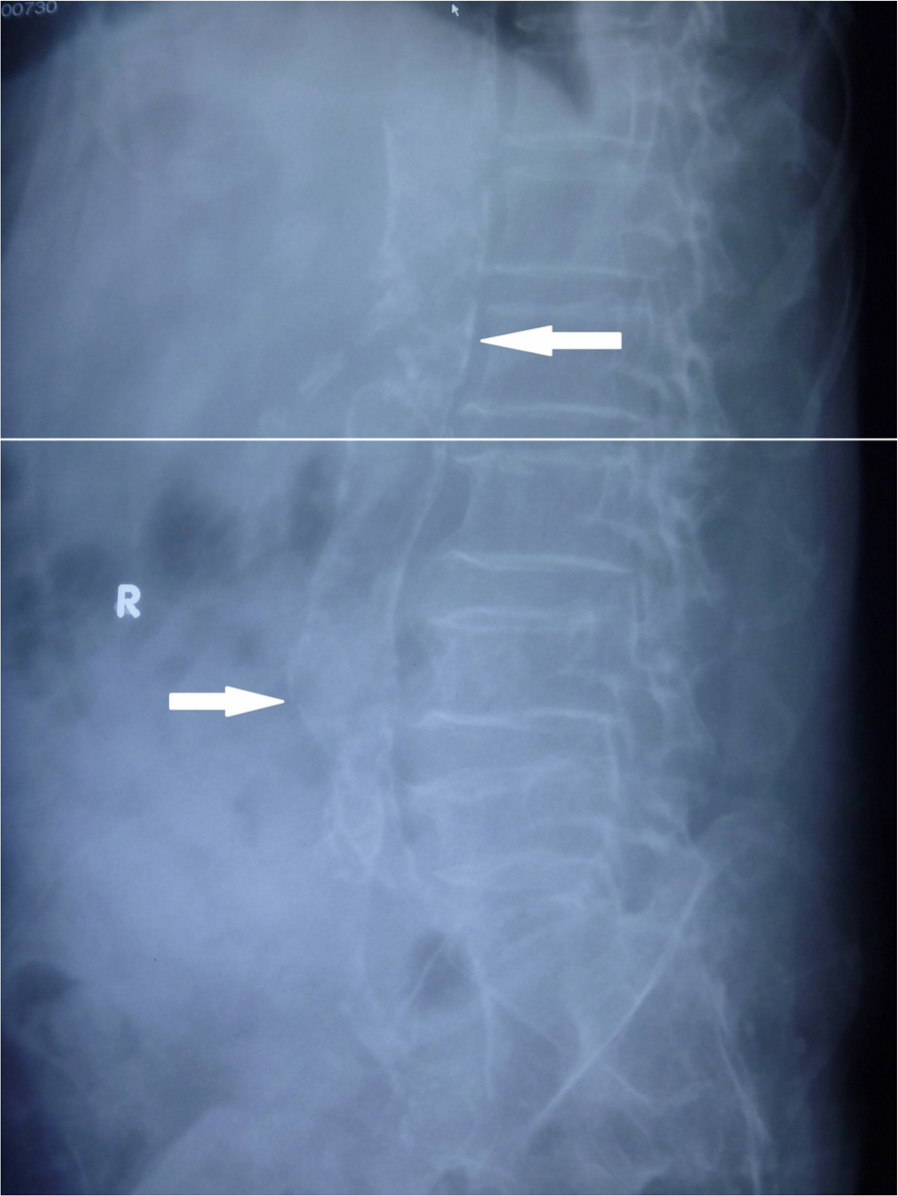
Vascular calcification in an abdominal radiograph.

The radiographs (computed radiography XG-1, Fuji, Japan) were reviewed by an independent radiologist. The pelvic radiographic films were divided into four sections by two lines: a horizontal line over the upper limit of both femoral heads and a median vertical line over the vertebral column. The films of each hand were divided by a horizontal line over the upper limit of the metacarpal bones [15]. The lateral abdominal radiographs were divided into two sections by a horizontal line over the intervertebral space between L2 and L3. The presence of linear calcifications in each section was counted as 1 and their absence was counted as 0. The final score was taken as the sum of all sections, and ranged from 0 to 10. VCs were evaluated in the iliac, femoral, radial digital and abdominal aortic arteries (Figures 1, 2 and 3).

### Covariates

Data regarding the demographic characteristics, history of diabetes, hypertension, stroke, hyperlipidemia, coronary artery disease, peripheral arterial disease and the duration of dialysis were collected from the records of patients in our Blood Purification Center. Blood pressures were measured before three dialysis sessions after a period of quiet supine rest using an Omron electronic sphygmomanometer. The average blood pressures and pulse pressures were then calculated.

10 ml of blood sample was drawn before starting a dialysis session for pre-dialysis measurements and 4 ml of blood sample was drawn at the end of dialysis session for post-dialysis measurements. Biochemical variables were measured using the Mindray BS-420 automatic biochemical analyzer. Pre-dialysis serum concentrations of creatinine, blood urine nitrogen, alkaline phosphatase, albumin, total cholesterol, triglycerides, calcium and phosphorus and post-dialysis concentrations of blood urine nitrogen were measured using routine laboratory methods. Kt/V was utilized to evaluate the adequacy of hemodialysis. Pre-dialysis parathyroid hormone (PTH) and ferritin (FER) levels were detected by chemiluminescence using a Siemens IMMULITE 1000 fully-automated chemiluminescence immunoassay analyzer with Siemens ancillary reagents. Pre-dialysis hemoglobin was measured using a SLS hemoglobin detection method with a Mindray BC5500 automated blood cell analyzer. Mean values of 3 measurements of serum calcium, phosphorus, albumin, alkaline phosphatase, and hemoglobin were calculated, other parameters were single measurements and acquired at the beginning of the study. Hyperlipidemia was defined as a total cholesterol level >200 mg/dL, a triglyceride level >150 mg/dL or the use of any lipid-lowering medication.

### Outcomes

Patient deaths during the study period were determined from a review of the Sichuan Provincial People’s Hospital’s data according to the CNRDS database (http://www.cnrds.net) or records at the Blood Purification Center. These included deaths that occurred in hospital or outside hospital. Cardiovascular mortality was defined as death due to myocardial infarction, heart failure, sudden cardiac death, or stroke. All-cause and cardiovascular mortality rates were calculated.

### Statistical methods

Baseline characteristics were compared between patients who survived or died during the follow-up period. Descriptive statistics are presented as the mean ± standard deviation or median with 25th-75th percentile. Differences in the mean and median values between groups were evaluated using the independent *t*-test and the Mann–Whitney *U* test, respectively. Categorical data was compared between groups with the chi-square test. Covariates with P-values <0.10 in the univariate analysis and with biological plausibility were included in our multivariate models. Survival curves were estimated with the Kaplan-Meier method and evaluated using the log-rank test to determine the difference in survival rates between groups with or without different VCs. Independent hazard ratios of all-cause and cardiovascular mortality associated with different VCs and different combinations of VCs were analyzed by the Cox proportional hazard regression for four different models. Model 1 was adjusted for demographic variables (age and gender); model 2 was adjusted for traditional cardiovascular risk factors (age, CAD, diabetes, hyperlipidemia and hypertension); model 3 was adjusted for dialysis specific factors (age, phosphorus level, Kt/V, albumin level, PTH level and duration of dialysis); and model 4 included covariates with P-values <0.10 in the univariate analysis (age, diabetes, phosphorus level, albumin level, hypertension, Kt/V, and pulse pressure). IBM SPSS statistical software (version 19.0, SPSS Inc., Chicago, IL, USA) was used to analyze all the data.

## Results

The average age of the participants was 60 ± 16 years, and 49.8% were men. The prevalence of VC was 70.0% (152 patients), and its prevalence in the abdominal aorta, iliac artery, femoral artery, digital artery and radial artery were 63.1%, 34.1%, 16.6%, 7.8% and 19.8% respectively (Table 1). Among the 152 patients with VC, only 15 patients (approximately 10%) did not have calcification of the abdominal aorta. During the follow-up period of 26 ± 7 months, 37 patients (17.1%) died, of whom 23 patients died of cardiovascular disease.

**Table 1:** Baseline characteristics of participants

**Variables**	**All (n = 217)**	**Alived (n = 180)**	**Deceased (n = 37)**	**P**
Age (y)	60 ± 16	57 ± 15	72 ± 10	<0.001
Male (%)	49.8	47.8	59.5	0.20
Cardiovascular disease (%)	12.9	12.2	16.2	0.51
Cerebral vascular disease (%)	7.4	6.1	13.5	0.12
History of hypertension (%)	88.5	90.5	81.1	0.10
Diabetes mellitus (%)	27.6	24.4	43.2	0.02
Dialysis vintage (m)	46 ± 41	46 ± 41	47 ± 43	0.89
Hyperlipidemia (%)	57.1	58.1	54.1	0.65
SBP (mm Hg)	144.7 ± 19.3	144.5 ± 18.8	145.2 ± 21.7	0.86
DBP (mm Hg)	76.1 ± 11.4	77.3 ± 11.0	70.4 ± 11.5	0.001
PP (mm Hg)	68.5 ± 17.3	67.2 ± 17.0	74.8 ± 17.8	0.02
Calcium (mmol/L)	2.22 ± 0.23	2.21 ± 0.23	2.26 ± 0.25	0.25
<2.10 (%)	31.5	32.4	27.0	
2.10-2.37 (%)	46.8	47.5	43.2	
>2.37 (%)	21.8	20.1	29.7	0.43
Phosphorus (mmol/L)	1.81 ± 0.59	1.81 ± 0.57	1.78 ± 0.64	0.78
<1.13 mmol/L (%)	10.2	8.4	18.9	
1.13-1.78 mmol/L (%)	44.4	45.8	37.8	
>1.78 mmol/L (%)	45.4	45.8	43.2	0.15
PTH (pg/ml)	286(174–598)	301(185–603)	227.0(107–516)	0.20
<150 (%)	20.8	19.0	29.7	
150-300 (%)	30.1	30.7	27.0	
>300 (%)	49.1	50.3	43.2	0.34
ALP (U/L)	103.72 ± 58.35	101.85 ± 57.76	112.81 ± 61.13	0.30
Albumin (g/L)	40.0 ± 3.1	40.3 ± 2.8	38.3 ± 3.9	0.01
Kt/V	1.34 ± 0.34	1.36 ± 0.35	1.20 ± 0.22	0.01
Hb (g/L)	105.94 ± 20.81	105.68 ± 22.06	104.35 ± 21.82	0.74
Calcification score	2(0–3)	1(0–3)	4(2–6)	<0.001
Aorta calcification (%)	63.1	57.8	89.2	<0.001
Iliac artery calcification (%)	34.1	28.9	59.5	<0.001
Femoral artery calcification (%)	16.6	12.2	37.8	<0.001
Digital artery calcification (%)	7.8	5.6	18.9	0.01
Radial artery calcification (%)	19.8	17.8	29.7	0.10

Abbreviations: *SBP* Systolic blood pressure, *DBP* Diastolic blood pressure, *PP* Pulse pressure, *PTH* Parathyroid hormone, *ALP* Alkaline phosphatase, *Hb* Hemoglobin.

The patients that died were older (72 ± 10 vs. 57 ± 15 years, P < 0.001), more often diabetic (43.2% vs. 24.4%, P = 0.02), had lower serum albumin levels (38.3 ± 3.9 g/L vs. 40.3 ± 2.8 g/L, P = 0.01), lower diastolic blood pressure levels (70.4 ± 11.5 mmHg vs. 77.3 ± 11.0 mmHg, P = 0.01), higher pulse pressures (74.8 ± 17.8 vs. 67.2 ± 17.0 mmHg, P = 0.02), and lower Kt/V (1.20 ± 0.22 vs. 1.36 ± 0.35, P = 0.01) compared to patients who survived during the follow-up period. They also had a higher prevalence of different VCs and higher overall VC scores (Table 1).

**Figure 4:**
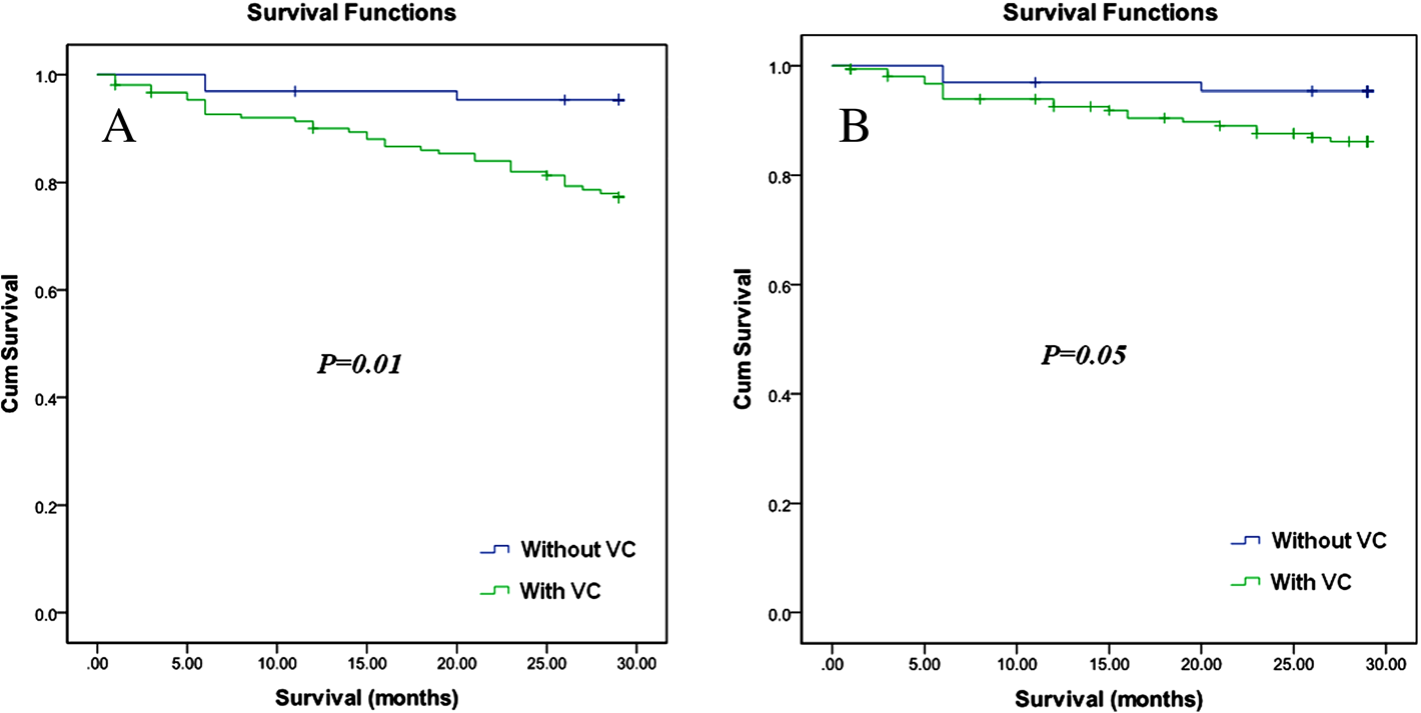
**Kaplan-Meier curves for patients with and without vascular calcification. A**) All-cause mortality; **B**) cardiovascular mortality.

**Figure 5:**
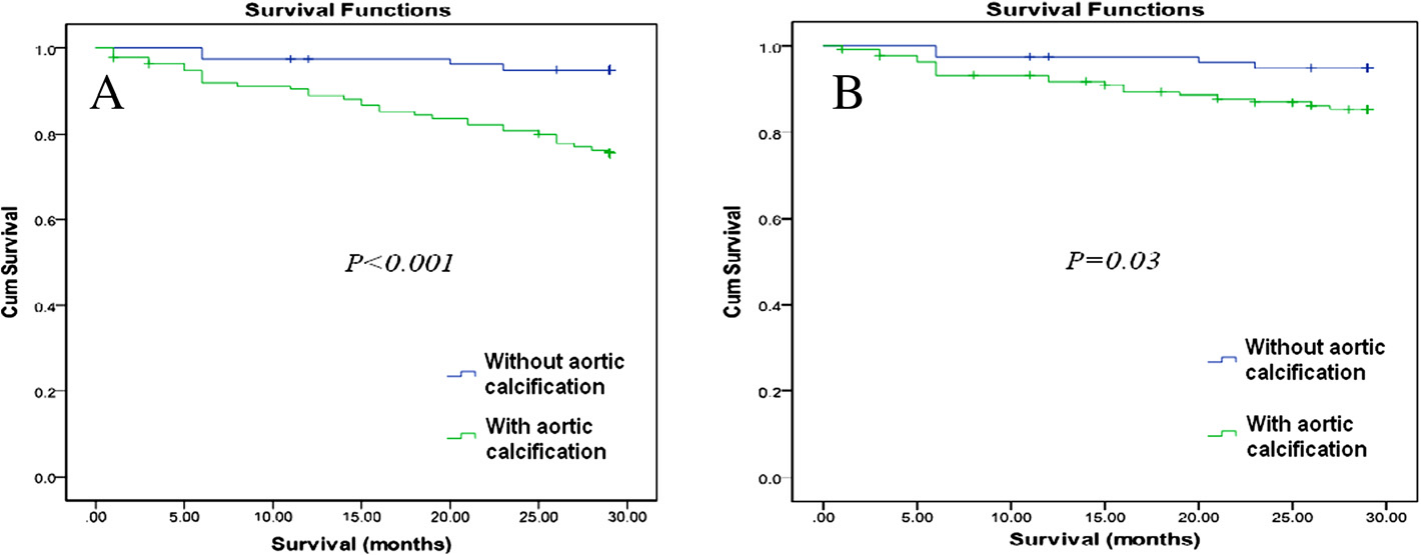
**Kaplan-Meier curves for patients with and without abdominal aortic calcification. A**) All-cause mortality; **B**) cardiovascular mortality.

Participants with VCs in different areas were at a greater risk of death (all-cause or cardiovascular) in the unadjusted analysis (Figures 4 and 5) with an increasing trend with increasing VC scores (Table 2). In the Cox regression (Tables 3 and 4), the VC score was independently associated with all-cause and cardiovascular mortality in models 2 and 3 (adjusted for cardiovascular risk factors [HR, 1.23; 95%CI, 1.09-1.39 and HR, 1.24; 95%CI, 1.07-1.43] and dialysis-related risk factors [HR, 1.26; 95%CI, 1.11-1.43 and HR, 1.30; 95%CI, 1.11-1.51], respectively).

**Table 2:** Univariate analysis of risk for all-cause and cardiovascular mortality

**Variable**	**HR for all-cause mortality (95%CI)**	**P-value**	**HR for cardiovascular mortality (95%CI)**	**P-value**
Age (per 1-yr older)	1.08(1.05-1.11)	<0.001	1.06(1.03-1.10)	0.01
Male (compared to female)	1.55(0.80-2.98)	0.19	1.14(0.51-2.59)	0.75
History of cardiovascular disease	1.26(0.53-3.02)	0.61	0.97(0.29-3.25)	0.95
History of cerebral vascular disease	2.03(0.79-5.22)	0.18	1.92(0.57-6.47)	0.29
Dialysis vintage (per 3-m longer)	1.00(0.98-1.03)	0.90	1.00(0.97-1.03)	0.89
Diabetes mellitus	2.13(1.11-4.07)	0.02	3.00(1.32-6.80)	0.01
Phosphorus^a^				
<1.13 mmol/L	2.38(0.96-5.89)	0.06	2.81(1.02-7.73)	0.05
>1.78 mmol/L	1.11(0.54-2.27)	0.78	0.68(0.26-1.79)	0.44
Calcium^b^				
<2.10 mmol/L	0.92(0.42-2.03))	0.84	0.883(0.32-2.43)	0.81
>2.37 mmol/L	1.54(0.72-3.32)	0.27	1.55(0.59-4.08)	0.37
PTH^c^				
<150 pg/ml	1.67(0.71-3.93)	0.24	1.51(0.49-4.67)	0.48
>300 pg/ml	1.01(0.46-2.22)	0.9	1.16(0.43-3.12)	0.78
ALP	1.00(1.00-1.01)	0.27	1.00(1.00-1.00)	0.27
Hyperlipidemia	0.87(0.46-1.67)	0.68	1.15(0.50-2.66)	0.74
Albumin (per 1 g/L increase)	0.83(0.75-0.92)	<0.001	0.79(0.70-0.90)	<0.001
Hemoglobin (per 1 g/L increase)	1.00(0.98-1.01)	0.55	1.00(0.98-1.01)	0.55
History of hypertension	0.46(0.20-1.05)	0.06	0.39(0.14-1.05)	0.06
Kt/V (per 1 increase)	0.18(0.06-0.59)	0.01	0.241(0.05-1.08)	0.06
Presence of aorta calcification	5.33(1.89-15.04)	0.01	3.03(1.03-8.93)	0.04
Presence of iliac artery calcification	3.15(1.64-6.08)	0.01	2.76(1.21-6.29)	0.02
Presence of femoral artery calcification	3.66(1.88-7.12)	0.01	2.61(1.15-5.92)	0.02
Presence of digital artery calcification	3.43(1.51-7.83)	0.01	5.03(1.98-12.76)	0.01
Presence of radial artery calcification	1.87(0.92-3.78)	0.08	1.92(0.79-4.66)	0.15
Presence of iliac or femoral artery calcification	3.05(1.57-6.00)	0.01	2.85(1.24-6.60)	0.01
Presence of radial or digital artery calcification	2.15(1.10-4.23)	0.03	2.52(1.09-5.83)	0.03
Vascular calcification score (per 1 score increase)	1.28(1.16-1.42)	<0.001	1.29(1.14-1.47)	<0.001
Pulse pressure (per 1 mmHg increase)	1.02(1.00-1.04)	0.01	1.22(1.00-1.04)	0.09

^a^ Compared with a reference group of 1.13-1.78 mmol/L; ^b^ compared with a reference group of 2.10-2.37 mmol/L; ^c^ compared with a reference group of 150–300 pg/ml.

**Table 3:** Relationship between vascular calcifications and all-cause mortality in hemodialysis patients

	**VC score**^**a**^	**Abdominal radiograph**	**Pelvic radiography**			**Hand radiographs**			**Ac**	**A+P**^**c**^	**A+P+H**^**c**^
		**Aorta VC**^**b**^	**All**^**b**^	**Iliac VC**^**b**^	**Femoral VC**^**b**^	**All**^**b**^	**Digital VC**^**b**^	**Radial VC**^**b**^			
Model 1	1.24(1.10-1.40)	3.27(1.15-9.30)	1.91(0.96-3.79)	1.97(1.00-3.88)	2.33(1.18-4.57)	1.98(1.01-3.90)	3.32(1.44-7.67)	1.83(0.90-3.74)	2.76(0.74-10.23)	2.94(0.80-10.78)	6.76(1.86-24.55)
Model 2	1.23(1.09-1.39)	4.69(1.60-13.69)	1.88(0.94-3.76)	1.99(1.00-3.96)	2.23(1.11-4.45)	1.50(0.72-3.13)	2.68(1.11-6.50)	1.35(0.63-2.88)	3.81(0.98-14.83)	3.90(1.02-14.88)	6.14(1.66-22.67)
Model 3	1.26(1.11-1.43)	3.38(1.18-9.69)	2.23(1.07-4.66)	2.24(1.09-4.60)	3.22(1.55-6.70)	2.06(1.02-4.20)	3.37(1.43-7.91)	1.79(0.85-3.77)	2.65(0.70-10.00)	2.96(0.80-10.99)	7.48(1.97-28.45)
Model4	1.25(1.10-1.41)	4.47(1.55-12.92)	2.32(1.11-4.82)	2.34(1.15-4.78)	3.09(1.49-6.42)	1.60(0.76-3.37)	2.62(1.04-6.58)	1.36(0.63-2.95)	4.38(1.09-17.58)	4.35(1.12-16.90)	7.43(2.01-27.44)

Notes: ^a^ Total vascular calcification score per 1 point score increase; ^b^ vascular calcification present in either artery; ^c^ vascular calcification present in abdominal radiographs, in both abdominal and pelvic radiographs, and in all radiographs (abdominal, pelvic and bilateral hand radiographs) compared to participants without any vascular calcifications in all radiographs.

Model 1: adjusted for age and gender.

Model 2: adjusted for age, CAD, hypertension, diabetes and hyperlipidemia.

Model 3: adjusted for age, phosphorus level, Kt/V, albumin level, PTH level and duration of dialysis.

Model 4: adjusted for age, diabetes, phosphorus level, albumin level, hypertension, Kt/V and pulse pressure.

Abbreviations: *A* Abdominal radiograph, *A+P* Abdominal and pelvic radiograph, *A+P+H* Abdominal, pelvic and bilateral hand radiographs.

**Table 4:** Relationship between vascular calcifications and cardiovascular mortality in hemodialysis patients

	**VC score**^**a**^	**Abdominal radiograph**	**Pelvic radiograph**			**Hands radiographs**			**A**^**c**^	**A+P**^**c**^	**A+P+F**^**c**^
		**Aorta VC**^**b**^	**All**^**b**^	**Iliac VC**^**b**^	**Femoral VC**^**b**^	**All**^**b**^	**Digital VC**^**b**^	**Radial VC**^**b**^			
Model 1	1.25(1.08-1.45)	1.97(0.66-5.88)	1.85(0.77-4.43)	1.79(0.76-4.21)	2.50(1.05-5.91)	2.30(0.99-5.32)	4.52(1.76-11.60)	1.88(0.77-4.60)	1.91(0.48-7.70)	1.09(0.24-5.08)	5.53(1.46-21.00)
Model 2	1.24(1.07-1.43)	3.12(0.99-9.79)	1.92(0.80-4.61)	1.94(0.82-4.59)	2.36(0.98-5.70)	1.54(0.64-3.70)	3.43(1.27-9.28)	1.21(0.48-3.02)	2.86(0.65-12.60)	1.70(0.34-8.39)	5.01(1.30-19.29)
Model 3	1.30(1.11-1.51)	2.01(0.66-6.13)	2.42(0.94-6.25)	2.24(0.90-5.58)	3.60(1.42-9.14)	2.28(0.94-5.54)	5.50(2.04-14.85)	1.64(0.64-4.22)	1.68(0.41-6.93)	1.29(0.27-6.26)	6.08(1.47-25.09)
Model 4	1.27(1.10-1.48))	2.86(0.93-8.81)	2.56(1.00-6.60)	2.39(0.96-5.93)	3.06(1.39-9.34)	1.64(0.67-4.00)	4.11(1.44-11.77)	1.20(0.47-3.06)	3.07(0.68-13.80)	2.06(0.41-10.40)	6.20(1.59-24.14)

Notes: ^a^ total vascular calcification score per 1 point score increase; ^b^ vascular calcification present compared to absent in either artery; ^c^ vascular calcification present in abdominal radiograph, in both abdominal and pelvic radiographs, and in all radiograph (abdominal, pelvic, forearm and hand radiographs) compared to participants without any vascular calcifications in all radiographs.

Model 1: adjusted for age and gender.

Model 2: adjusted for age, CAD, hypertension, diabetes and hyperlipidemia.

Model 3: adjusted for age, phosphorus level, Kt/V, albumin level, PTH level and duration of dialysis.

Model 4: adjusted for age, diabetes, phosphorus level, albumin level, hypertension, Kt/V and pulse pressure.

Abbreviations: *A* Abdominal radiograph, *A+P* Abdominal and pelvic radiograph, *A+P+F* Abdominal, pelvic and hand radiographs.

In the Cox regression analysis (Tables 3 and 4), abdominal aortic VCs were associated with all-cause mortality in model 2 adjusted for cardiovascular risk factors (HR, 4.69; 95%CI, 1.60-13.69) and in model 3 adjusted for dialysis-related factors (HR, 3.38; 95%CI, 1.18-9.69), but were not significantly associated with cardiovascular mortality in all models. VCs observed in pelvic radiographs (iliac artery or femoral artery calcification) were associated with all-cause mortality in model 3 (HR, 2.23; 95%CI, 1.07-4.66). VCs observed in the hand radiographs (digital artery or radial artery calcifications) were associated with all-cause mortality in model 3 (HR, 2.06; 95%CI, 1.02-4.20). VCs observed in the pelvic or hand radiographs were not significantly associated with cardiovascular mortality. Overall, digital artery calcifications remained significantly associated with all-cause or cardiovascular mortality in all four models.

In order to study the different contribution to patient mortality by VCs in different regions, we calculated the frequency of different combinations of the above three radiographs; combinations with a frequency of less than 15 were excluded to avoid over-specification. As a result of the high prevalence of abdominal aorta calcifications, only three combinations were ultimately selected. These were as follows: (A) VC in the abdominal radiograph (n = 53 patients); (A+P) VC present in both the abdominal and pelvic radiographs (n = 46 patients); and (A+P+H) VC present in abdominal, pelvic and hand radiographs (n = 26 patients). When the three combinations were each entered into the models as a categorical variable (Tables 3 and 4), compared with patients without VC, ‘A’ was only significant for all-cause mortality in model 4, VC observed in ‘A+P’ was associated with all-cause mortality in cardiovascular risk factors adjusted model (model 2) and in model 4, while ‘A+P+H’ was significantly associated with an increase in all-cause mortality of more than 6-fold and an increase in more than 5-fold for cardiovascular mortality compared to patients without VCs in any of the radiographs. ‘A’ or ‘A+H’ VCs were not significantly associated with increased cardiovascular death in this study.

## Discussion

The prevalence of VC in our study was 70.0%, and although there were some differences from previous studies because of the different evaluation methods used, the finding of a high prevalence of VC in hemodialysis patients was consistent with most studies [8,15,16]. Our study showed an increased risk of all-cause and cardiovascular mortality with higher VC scores, which was consistent with most previous studies [15,17-19]. Our results showed a positive correlation between all-cause mortality and abdominal aortic VC, after adjustment for different VC combinations. This was only significant for the cardiovascular risk factor adjusted model and the integrated model, and no significant results were found with regards to cardiovascular mortality. VC present in ‘A+P’ was significantly associated with all-cause mortality only in the model adjusted for cardiovascular risk factors, while ‘A+P+H’ VCs showed significantly higher hazard ratios (HR) for all-cause or cardiovascular mortality in all models (5-fold and 4-fold, respectively, compared to patients without VC). This could have resulted from the additive effect of an increasing VC score, or the independent effect of the additional VCs observed in the radiographs of the hands.

The prevalence of aortic calcification has been reported to be more than 50% in CKD patients [5,6,11], which is in accordance with our results. This may be associated with several risk factors, including increasing age [12], genetic factors [20] and body composition [21] in addition to traditional cardiovascular risk factors and CKD/dialysis-related risk factors. Therefore, this may decrease its ability to distinguish disease related status that could cause poor outcomes from the effect of natural aging, or its clinical value might be overestimated when effects of other coexisting VCs are included. In our center, we did not find a constant positive association between abdominal aortic calcification and all-cause or cardiovascular mortality in all models after adjusting for VC present in other radiographs combinations, while a significant association between all-cause and cardiovascular mortality and VC present in all three radiographic sites was observed. Although the additive risk may be due to the total VC score, the probability of an independent effect of VC observed in hand radiographs cannot be excluded. It follows from the above that if abdominal aortic calcification is solely analyzed without coexisting VCs, the risk for patient death would be overestimated. Many studies have revealed a significant association between aortic calcification and patient mortality [5,6,22-24], but few studies have focused on the independent effect on patient survival of VCs in different anatomical sites. A more accurate method of measuring the systemic burden of VCs could be developed to optimize risk stratification in high-risk dialysis patients [6].

We did not find a significant association between patient mortality and radial artery calcification in either the univariate or multivariate analysis. We have little insight into the relationship between radial artery calcification and patient death, with the exception of a single study by Schlieper et al. [25]. They reported an increased risk (HR, 2.15; 95%CI, 1.05-4.39, P = 0.036) for patient mortality with vascular access calcification after their analysis was adjusted for age, diabetes, duration of dialysis and pre-existing vascular disease. However, they also reported a positive correlation between the presence of other VCs (femoral/iliac) and vascular access calcification, which makes it more difficult to distinguish between the independent influences on patient outcome by different VCs. Risk factors for radial artery calcification are more complicated than for other VC sites, including the effects of arteriovenous fistula (AVF) construction, hemodynamic factors and inadequate dialysis [26-28]. Although we found a lower prevalence of radial artery calcification than AVF calcification by CT fistulogram [28], we could not include all of the visible calcification of the radial artery (including the vessels used for AVF) because of the limitations of hand radiography which included only part of the forearm. This may partly explain the inconsistent findings in our study. Calcification of the radial artery may lead to the failure of vascular access maturation [29]. Moreover, digital artery calcification and the presence of VC in hand radiographs (whether adjusted for other VCs or not) was significantly associated with all-cause and cardiovascular mortality. Therefore, hand radiographs may be conducted before AVF surgery to avoid subsequent failure as a result of fistula blockages. It could be an investigative method for identifying patients at high risk of death. Further studies need to be performed to confirm this finding and to compare it with coronary artery calcification, the gold standard VC for patient outcomes.

VCs in the femoral or iliac arteries was correlated with an increased risk of all-cause mortality in both the univariate and multivariate analysis. Iliac artery calcification was significantly associated with all-cause mortality, while it was not significantly associated with cardiovascular mortality in the multivariate analysis in our study, which was consistent with previous research in a large population with longer follow-up durations [30]. Moreover, iliac vessel calcification was reported to be associated with mortality in renal transplant patients [31], and the plain X-ray of the pelvis was a useful screening tool to identify patients requiring further detailed vascular imaging before transplantation. This could be of value in choosing the ‘landing site’ for implantation or to exclude patients not suitable for transplantation. Therefore, it would make sense if pelvic radiography was carried out as a screening method for hemodialysis patients at high risk of death from all causes or those on the waiting list for renal transplantation.

There were several limitations in our study. Firstly, the study sample was relatively small. The number of VCs present in different combinations, especially those without abdominal aortic calcification, was relatively small to distinguish the effect on patient mortality of different groups. Secondly, the semi-quantification of VCs was relatively simple, but since VC was not quantitated in specific arteries, important information may have been missed. Thirdly, other types of VC were not evaluated in this study because of the difficulty in differentiating intimal calcification from tunica media calcification by plain X-rays, and this has previously been shown to be relevant [32]. Finally, the follow-up period was relatively short compared to similar studies, and with the limitation of retrospective studies including incomplete information about comprehensive treatment and compliance, our findings should be confirmed in future prospective observational studies.

The strength of the study is its ability to provide comparative information concerning the associations of VC at different arterial sites with all cause and cardiovascular mortality. We found that abdominal aortic calcification alone was not a good predictor of patient mortality in our population, which differed from most previous studies, but a strong association was found between mortality and concurrent VC in the abdomen, pelvis and hands. And it should stimulate expansion of this focus.

## Conclusions

In summary, VC is prevalent in hemodialysis patients and is associated with all-cause and cardiovascular mortality. VCs in different arteries carry different mortality-related risks. Abdominal aorta calcification is highly prevalent, which may decrease its prognostic value for the survival of hemodialysis patients. In this group of HD patients, with a short follow-up, abdominal vascular calcification was highly prevalent but was not shown to be a superior prognostic tool over other vascular area evaluated. VCs present on hand radiographs appear to be more closely associated with patient mortality, but this needs to be confirmed by future studies with larger sample sizes and different combinations of VC sites.

## Abbreviations

VC: Vascular calcification; CVD: Cardiovascular disease; AVF: Arteriovenous fistula.

## Competing interests

The authors declare that they have no competing interests.

## Authors’ contributions

HDQ and WSK collected and processed the data, helped with the study design and drafted the manuscript. PL, WF, WJR, WZT, ZY, DF and GH collected the data. LGS and HQ participated in the study design and coordination, and performed the statistical analysis. WL conceived the study, participated in its design and coordination, performed the statistical analysis and helped to draft the manuscript. All authors have read and approved the manuscript.

## Pre-publication history

The pre-publication history for this paper can be accessed here:

http://www.biomedcentral.com/1471-2369/14/120/prepub
